# Impact of malocclusion on the quality of life of children aged 8 to 10 years

**DOI:** 10.1590/2177-6709.23.2.046-053.oar

**Published:** 2018

**Authors:** Sônia Rodrigues Dutra, Henrique Pretti, Milene Torres Martins, Cristiane Baccin Bendo, Miriam Pimenta Vale

**Affiliations:** 1 Private practice (Belo Horizonte/MG, Brazil).; 2 Universidade Federal de Minas Gerais, Faculdade de Odontologia, Departamento de Odontopediatria e Ortodontia (Belo Horizonte/MG, Brazil).; 3 Universidade Estadual de Montes Claros, Curso de Odontologia (Montes Claros/MG, Brazil).

**Keywords:** Mixed dentition, Malocclusion, Quality of life.

## Abstract

**Objective:**

The aim of the present cross-sectional study was to assess the impact of malocclusion on the quality of life of children aged 8 to 10 years attending public elementary schools in Belo Horizonte, State of Minas Gerais, Brazil.

**Methods:**

The Brazilian version of the Child Perceptions Questionnaire 8-10 (CPQ8-10) was used to evaluate oral health-related quality of life. The children were examined for the diagnosis of malocclusion using the Dental Aesthetic Index (DAI). The data were analyzed by bivariate and multivariate descriptive statistics using Poisson regression at a 5% significance level. A total of 270 children participated in the study.

**Results:**

Children with normal occlusion or mild malocclusion (DAI ≤ 25) were 56% less likely (95%CI: 0.258-0.758; *p*= 0.003) to have their quality of life affected compared with children diagnosed with extremely severe malocclusion (DAI ≥ 36). Children with a maxillary anterior overjet ≥ 3 mm had higher CPQ_8-10_ mean scores (19.4; SD = 17.1) than those with an overjet < 3 mm (13.6; SD = 11.7; *p*= 0.038).

**Conclusions:**

Extremely severe malocclusion and pronounced maxillary anterior overjet were associated with a negative impact on quality of life.

## RESUMO

**Objetivo:**

o objetivo do presente estudo transversal foi avaliar o impacto da má oclusão na qualidade de vida de crianças na faixa etária de 8 a 10 anos de idade, estudantes de escolas da rede pública da cidade de Belo Horizonte, estado de Minas Gerais, Brasil.

**Métodos:**

o instrumento utilizado para medir a qualidade de vida relacionada à saúde bucal foi a versão brasileira do Child Perceptions Questionnaire 8-10 (CPQ_8-10_). As crianças foram examinadas para diagnóstico de má oclusão utilizando-se o Dental Aesthetic Index (DAI). A análise dos dados foi realizada por meio de estatística descritiva, bivariada e multivariada por meio da regressão de Poisson, com nível de significância de 5%. Participaram do estudo 270 crianças.

**Resultados:**

crianças com oclusão normal ou má oclusão leve (DAI ≤25) apresentaram 56% menos probabilidade (IC 95%: 0,258-0,758; *p*= 0,003) de impacto na qualidade de vida, comparadas com crianças diagnosticadas com má oclusão muito grave (DAI ≥ 36). Crianças com sobressaliência superior anterior ≥ 3 mm apresentaram maiores escores médios de CPQ_8-10_ (19,4; DP = 17,1) do que aqueles com sobressaliência < 3 mm (13,6; DP=11,7; *p*= 0,038).

**Conclusões:**

a má oclusão muito grave e a sobressaliência superior anterior aumentada se associaram ao impacto negativo na qualidade de vida.

## INTRODUCTION

Quality of life is a concept that includes several domains, such as the subjective perception of physical, psychological, and social functions, in addition to a subjective sense of well-being.^1^ ^,^ ^2^ Oral health is crucial for good quality of life^3^ as it may have an impact on children’s feeding, smiling, speaking, and socializing. Facial appearance influences self-esteem and emotional well-being, playing an important role in social interaction. Changes in these functions will consequently affect children’s quality of life.^4^

Several authors have investigated the benefits of using more subjective criteria, such as an individual’s perception of his/her health status and the impact of his/her disease on his/her quality of life, in the clinical assessments made by healthcare professionals. Clinical measures are important to determine a patient’s normative need for treatment and associating them with information on the impact of oral changes in people’s daily lives turns out to be useful.^2^ ^,^ ^5^ ^-^ ^8^

Normative indices - which are quite useful for the planning of public health services in orthodontics - have been used for assessing the severity of malocclusion. These indices allow determining the priority of care among extremely severe cases by the Brazilian Unified Health System, known as SUS.^9^

Malocclusion is a health problem that has received rapt attention, being the third most prevalent oral disease, outranked only by dental caries and periodontal disease.^9^ Because of its high prevalence, malocclusion is regarded as a public health problem that may negatively interfere in patients’ quality of life, hinder their social interaction, and affect their psychological well-being.^8^ The same type of malocclusion has different psychosocial impacts.^10^ ^,^ ^11^

Numerous studies have assessed the impact of malocclusion on quality of life.^12^ ^-^ ^17^ However, there is a paucity of studies on the impact of malocclusion on mixed dentition, especially studies that address the psychosocial factors that lead people to seek orthodontic treatment.^12^ Most studies on the impact of malocclusion on quality of life have assessed adolescents and young adults.^14^ ^-^ ^17^

Previous studies involving children aged 8 to 10 years revealed that those children with malocclusion were more prone to have a negative impact on their quality of life than malocclusion-free individuals.^12^ ^,^ ^13^ Some types of malocclusion also had a higher impact on quality of life.^12,13^

Accordingly, the aim of this study was to contribute to the formulation of public health policies in orthodontics. To achieve such goal, the study assessed the impact of malocclusion on the quality of life of children aged 8 to 10 years enrolled in public schools of Belo Horizonte, State of Minas Gerais, Brazil

## MATERIAL AND METHODS

This cross-sectional study was conducted in Belo Horizonte, State of Minas Gerais, Brazil. A total of 270 children aged 8 to 10 years, of both sexes, were included in the study. Children with special needs or cognitive impairments reported by parents and teachers were excluded for the sample. Children with traumatic dental injuries or enamel development defects in permanent teeth were also excluded, as well as those who were wearing orthodontic appliances or with history of previous orthodontic treatment. 

All of the participants were regularly enrolled in the city’s public elementary schools, each of which belonged to one of the nine regional administrative units. Schools were selected from the list of all elementary schools in Belo Horizonte, provided by the State of Minas Gerais Department of Education. First, one public elementary school was randomly selected in each administrative district in Belo Horizonte. After that, the classes within the schools were selected. All 8-to-10-year-old students attending the selected classes were asked to participate. The sample was completed when the target number was reached.

The power of the sample was calculated based on the impact of malocclusion on quality of life, using EpiInfo software. The following parameters were used: 95% confidence interval; children with normal occlusion or mild malocclusion, n=157 (CPQ_8-10_ mean=13.5; SD=11.7); children with definite, severe and extremely severe malocclusion, n=113 (CPQ_8-10_ mean=20.4; SD=16.4). Using theses parameters, the power of the study was 96.9%.

The Brazilian version of the Child Perceptions Questionnaire 8-10 (CPQ_8-10_) was applied to assess oral health-related quality of life. The responses to the questionnaire follow the 5-point Likert scale, with scores 0 to 4 for each item. The sum of scores can vary from 0 to 100. Score zero (0) indicates no impact of children’s oral health on their quality of life, while Score 100 indicates the opposite, i.e., maximum impact of oral health on children’s quality of life.^18^

The clinical examination was performed with wooden spatulas, and CPI (Community Periodontal Index) periodontal probes. The CPI periodontal probes were used to measure occlusal characteristics in millimeters. The children were evaluated by one calibrated dentist, who used the Dental Aesthetic Index (DAI) for malocclusion and the DMFT/deft (decayed, missing and filled teeth/decayed, indicated for extraction, and filled teeth) for dental caries, both recommended by the World Health Organization (WHO).^19^ Oral examinations were performed in the classroom with the child sat on a chair near the window. 

Calibration for DAI (malocclusion) and DMFT/deft (dental caries) included theoretical and clinical stages. The theoretical stage consisted of discussions about the diagnostic criteria for malocclusion and dental caries by means of photographs and plaster models. The calibration process was coordinated by experts in pediatric dentistry and orthodontics as golden standard. 

The clinical stage was conducted with 20 children. Children were evaluated by the golden standard and the examiner. These children were not included in the total study sample. The same children were reexamined one month afterwards. The Cohen’s kappa coefficient for interrater reliability was 0.81 for dental caries and 0.90 for malocclusion, whereas for intrarater reliability, it was 0.87 for dental caries and 0.91 for malocclusion (agreement between the examiner and the gold standard). The coefficients showed good and excellent agreement.

DAI scores less than or equal to 25 indicate normal occlusion or mild malocclusion with little or no orthodontic treatment need. Scores between 26 and 30 indicate definite malocclusion requiring elective treatment. Scores between 31 and 35 indicate severe malocclusion with highly desirable treatment. Scores equal to or greater than 36 indicate extremely severe or handicapping malocclusion with mandatory treatment.

Dental caries was included in the clinical examination and treated as confounding variable; its assessment was based on the DMFT/deft indices. DMFT/deft was used as a quantitative variable. And for an additional statistical analysis, this variable was dichotomized according to the presence or absence of cavitation caused by dental caries.

The pilot study was undertaken in a school that did not participate in the main study so as to test the methods and understand the tools used for data collection. 

The Social Vulnerability Index (SVI) was used for the socioeconomic classification. The SVI is an area-based measure drafted for the city of Belo Horizonte and determines to what extent the population of each region of the city is vulnerable to social exclusion. The index is made up of five dimensions: environmental, cultural, economic, legal and security/survival. There are five different classes: Class I comprises the most socially vulnerable families and Class V comprises the least socially vulnerable families. In this study, the SVI was grouped into two categories for statistical purposes: Classes I and II were grouped in the category ‘high social vulnerability’, and Classes III to V were grouped in the category ‘low social vulnerability’. As children usually live near their schools and study in a social environment similar to that of their homes, school districts were used for this classification.^12^ ^,^ ^20^

The statistical analyses were made by Statistical Package for the Social Sciences (SPSS for Windows, version 22.0, SPSS Inc., Chicago, IL, USA). The Kolmogorov-Smirnov test demonstrated that CPQ_8-10_ scores were not normally distributed. The data were analyzed by descriptive analysis using absolute and relative frequencies, means, and standard deviation. Mann-Whitney and Kruskal-Wallis tests were used for comparison of CPQ_8-10_ means with the independent variables. Bivariate Poisson regression was used to compare the CPQ_8-10_ means between the DAI categories (DAI≤ 25; DAI = 26 to 30; DAI = 31 to 35; DAI ≥ 36). Poisson regression with robust error variance was used for the multivariate analysis. The variables were inserted into the regression model according to their statistical significance (*p*< 0.20). The significance level was set at 5%.

This study was approved by the Research Ethics Committee of *Universidade Federal de Minas Gerais* (protocol #40521114.9.0000.5149). Only those children who granted authorization and obtained it from their parents/legal guardians participated in the study. Parents/guardians and children read and signed an informed consent form prior to their participation in the study.

## RESULTS

A total of 270 children aged 8 to 10 years attending public schools in Belo Horizonte were included in the study. Fifty percent of the children (*n*= 135) were male and 67.8% had non-cavitated caries lesions. From these, 175 (58.1%) children had normal occlusion or mild malocclusion; 75 (27.8%) children presented with definite malocclusion (DAI = 26 to 30); 31 (11.5%) children were diagnosed with severe malocclusion (DAI = 31 to 35), and seven (2.6%) children had extremely severe malocclusion (DAI ≥ 36) (Table 1). 

**Table 1:** Sample frequency (n = 270) according to the variables; Belo Horizonte, Brazil, 2015.

Variables	Frequency n (%)
Sex	
Male	135 (50%)
Female	135 (50%)
Age (years)	
8	114 (42.2%)
9	109 (40.4%)
10	47 (17.4%)
Social vulnerability	
Low vulnerability	188 (69.6%)
High vulnerability	82 (30.4%)
Presence of untreated decayed teeth	
No	183 (67.8%)
Yes	87 (32.2%)
DMFT (mean and SD)	0.91 (1.49)
DAI	
≤ 25	157 (58.1%)
26-30	75 (27.8%)
31- 35	31 (11.5%)
≥ 36	07 (2.6%)

Note: SD = standard deviation; DMFT was used as a quantitative variable.

Table 2 shows the frequency of each type of malocclusion. The most frequent types of malocclusion were anterior crowding in one segment (39.6%), anterior diastema in one segment (44.1%), maxillary anterior overjet ≥ 3 mm (24.0%), and one-half cusp molar relationship (24.1%). 

**Table 2:** Frequency of the types of malocclusion; Belo Horizonte; Brazil, 2015.

Malocclusion	Frequency n (%)
Anterior crowding	
No crowding	96 (35.6%)
One crowded segment	107 (39.6%)
Two crowded segments	67 (24.8%)
Anterior spacing	
No spacing	51 (18.9%)
One segment with spacing	119 (44.1%)
Two segments with spacing	100 (37.0%)
Incisal diastema (mm)	
<2	227 (84.1%)
≥2	43 (15.9%)
Larger anterosuperior irregularity (mm)	
<2	212 (78.5%)
≥2	58 (21.5%)
Larger anteroinferior irregularity (mm)	
<2	213 (78.9%)
≥2	57 (21.1%)
Maxillary anterior overjet (mm)^1^	
<3	198 (73.3%)
≥3	65 (24.0%)
Mandibular anterior overjet (mm)	
Absent	247 (91.5%)
Present	23 (8.5%)
Anterior open bite (mm)	
Absent	250 (92.5%)
Present	20 (7.5%)
Anteroposterior molar relationship	
Normal	205 (75.9%)
One-half cusp	65 (24.1%)

Note: ^1^ Maxillary anterior overjet does not amount to 100% due to seven cases of anterior crossbite.

Table 3 displays the results for the bivariate analysis between the DAI categories and dental caries on the CPQ_8-10_ scores. Children without malocclusion or with mild malocclusion (DAI ≤ 25) were 56% less likely (95%CI: 0.258-0.758; *p*= 0.003) to have a negative impact on their quality of life, compared with those children diagnosed with extremely severe malocclusion (DAI ≥ 36). Presence of untreated dental caries was associated with negative impact on quality of life (*p*= 0.010).Higher DMFT index was associated with higher CPQ_8-10_ scores (*p*< 0.001). Social vulnerability was not associated with quality of life (*p*= 0.327).

**Table 3:** Bivariate Poisson regression showing the influence of DAI categories, dental caries and social vulnerability on quality of life; Belo Horizonte; Brazil, 2015.

CPQ_8-10_			
	Mean (SD)	PR (95%CI)	p value
DAI (Malocclusion)			
DAI≤ 25 (Normal occlusion or mild malocclusion)	13.5 (11.7)	0.442 (0.258-0.758)	0.003
DAI= 26-30 (Definite malocclusion)	18.1 (15.6)	0.591 (0.339-1.031)	0.064
DAI= 31 - 35 (Severe malocclusion)	12.5 (10.5)	0.407 (0.224-0.740)	0.003
DAI≥36 (Extremely severe malocclusion)	30.6 (23.2)	1	
Presence of untreated decayed teeth			
No	13.7 (12.9)	0.758 (0.613-0.937)	0.01
Yes	18.1 (14.1)	1	
DMFT	_______	1.11 (1.06-1.63)	<0.001
Social vulnerability			
Low vulnerability	14.6 (13.0)	0.892 (0.710-1.121)	0.327
High vulnerability	16.3 (14.4)	1	

Note: CI = confidence interval; DAI = Dental Aesthetic Index; PR = prevalence ratio; SD = standard deviation; DMFT was used as a quantitative variable.

Table 4 shows the CPQ_8-10_ mean values according to the independent variables. Children with maxillary anterior overjet ≥ 3 mm had higher CPQ_8-10_ means (19.4; SD = 17.1) than those with an overjet < 3 mm (13.6; SD = 11.7; *p*= 0.038). The other independent variables were not significantly associated with CPQ_8-10_ (*p*> 0.05).

**Table 4:** Mean and standard deviation of CPQ_8-10_ according to independent variables; Belo Horizonte; Brazil, 2015.

Variables	CPQ_8-10_	p value
	Mean (SD)	
Anterior crowding^2^		
No crowding	14.2 (12.6)	0.813
One crowded segment	15.0 (12.6)	
Two crowded segments	16.5 (15.8)	
Anterior spacing^2^		
No spacing	16.6 (15.8)	0.627
One segment with spacing	14.5 (13.2)	
Two segments with spacing	15.0 (12.5)	
Incisal diastema^1^		
<2	14.6 (13.5)	0.056
≥2	17.9 (13.1)	
Anterosuperior irregularity^1^		
<2	15.2 (13.8)	0.82
≥2	14.9 (12.2)	
Anteroinferior irregularity^1^		
<2	14.5 (13.4)	0.084
≥2	17.3 (13.5)	
Maxillary anterior overjet^1^		
<3mm	13.6 (11.7)	0.038
≥3mm	19.4 (17.1)	
Mandibular anterior overjet^1^		
Absent	15.2 (13.7)	0.866
Present	14.3 (10.6)	
Anterior open bite^1^		
Absent	14.7 (13.0)	0.27
Present	19.5 (18.3)	
Anteroposterior molar relationship^1^		
Normal	14.2 (12.4)	0.229
One-half cusp	18.0 (16.0)	

Note: ^1^ Mann-Whitney test; ^2^ Kruskal-Wallis test; SD = standard deviation.

Table 5 shows the results for the multivariate Poisson regression with robust error variance. The final model adjusted by incisal diastema, larger anteroinferior irregularity, social vulnerability and DMFT demonstrated that children with a maxillary anterior overjet ≥ 3 mm were 32% more likely (95% CI: 1.03-1.70; *p*= 0.028) to have a negative impact on their quality of life than those with a maxillary anterior overjet < 3 mm. 

**Table 5:** Multivariate Poisson regression showing the influence of types of malocclusion and dental caries on quality of life; Belo Horizonte; Brazil, 2015.

Malocclusion	PR	95% CI	p value
Incisal diastema (in mm)			
<2	1	0.88-1.48	0.313
≥2	1.14		
Larger anteroinferior irregularity (in mm)			
<2	1	0.93-1.49	0.176
≥2	1.18		
Maxillary anterior overjet (in mm)			
<3mm	1	1.03-1.70	0.028
≥3mm	1.32		
Social vulnerability			
Low vulnerability	1	0.90-1.42	0.296
High vulnerability	1.13		
DMFT	1.09	1.05-1.15	<0.001

Note: PR = prevalence ratio; CI = confidence interval; DMFT was used as a quantitative variable.

Presence of untreated decayed teeth variable was not inserted in the multivariate model due to its high correlation with DMFT.

## DISCUSSION

There is a growing interest among researchers in the influence of facial esthetics on the quality of life of children and adolescents. This study found that children with extremely severe malocclusion were more likely to have a negative impact on quality of life, corroborating the findings of other studies on the effect of malocclusion on quality of life conducted with children in the same age group (8 to 10 years).^12^ ^,^ ^13^

The bivariate analysis demonstrated that most types of malocclusion were not statistically associated with oral health-related quality of life. Studies report that tooth decay is associated with an impact on the quality of life,^3^ ^,^ ^12^ by this reason dental caries was included in the clinical examination and treated as confounding variable. However, dental caries did not interfere in the results of malocclusion. Of the ten occlusal characteristics assessed by DAI, the present study demonstrated that only accentuated maxillary anterior overjet was associated with a negative impact on quality of life. Other studies carried out with children aged 8 to 10 years detected more occlusal characteristics statistically associated with an impact on quality of life.^12,13^ One of this studies demonstrated that anterior segment spacing and anterior mandibular overjet were associated with worse quality of life.^12^ The other study found such association with upper anterior irregularity, anterior open bite and diastema.^13^ These differences may be due to sample size and studied population. The present study was conducted with 270 children only from public schools, and one of these previous studies was carried out with a larger sample size (n=1,204) from public and private schools.^12^ The other previous study was conducted with a smaller sample (n=102) only from public schools.^13^

The analysis of the results revealed that malocclusion (DAI >25) affected 41,9% of the children examined, while previous studies carried out with children of the same age group found that malocclusions affected 32,2%^12^ and 61%^13^ of the children examined. We should emphasize the difference in the sample size of the studies: 1,204 chidren,^12^ 102 children^13^ and 270 children in the present study. 

It was not possible to compare the present results with many others carried out with children of the same age group since there is a paucity of studies on the impact of malocclusion on the mixed dentition,^12^ specially studies that address the psychosocial factors that lead people to seek for orthodontic treatment. 

Previous studies described the presence of several occlusal characteristics associated with a negative impact on quality of life,^14^ ^-^ ^17^ including the presence of larger maxillary anterior overjet.^15^ ^,^ ^16^ ^,^ ^17^ However, these results must be viewed with caution as the age range of participants was different from the one used in the present study. These studies were conducted with adolescents and young adults. Adolescents^13^ tend to be more concerned with body image and it is important the approval from others of the same age group. Children^13^ aged eight to ten years old are more concerned with the approval of adults. It is important to highlight that most studies on the impact of malocclusion on oral health-related quality of life have been conducted with adolescents and young adults. 

The presence of malocclusion, mainly in the anterior region, may interfere with children’s and adolescents’ psychosocial well-being. DAI was the criterion for the diagnosis of malocclusion in this study as it uses a single score, being practical for epidemiologists,^21^ ^,^ ^22^ and assesses occlusal characteristics that could potentially cause psychosocial impairment.^22^

DAI was designed for permanent dentition. There is no orthodontic index that is specific to mixed dentition. DAI could thus overestimate the need for orthodontic treatment, and this tends to occur more often with mixed dentition because of transient occlusal changes such as midline diastema, spacing between incisors, edge-to-edge molar relationship, among others.^23^ Another transient change observed in mixed dentition is temporary primary crowding, which has spontaneous correction.^24^ Consequently, these transient changes in mixed dentition may get the DAI score up, classifying malocclusion into a severity level that is not necessarily present. This is one of the limitations of the present study. Moreover, cross-sectional studies have limitations inherent to the design.

A previous study asserts that malocclusion does not self-correct from deciduous to mixed dentition, nor from mixed to permanent dentition,^24^ thereby underscoring the importance of orthodontic assessment and diagnosis of children with mixed dentition. Therefore, it is paramount to make a distinction between transient changes and malocclusion at that stage. Early diagnosis aids with interceptive orthodontics and may prevent psychosocial distress in children with malocclusion.

An earlier study emphasized the importance of classifying malocclusion into severity levels in order to prioritize the treatment of more severe cases at healthcare units affiliated with SUS.^9^ It is also essential that the assessment of oral health-related quality of life be included among normative criteria.^17^ In the present study, the normative criterion for the diagnosis of malocclusion was combined with the children’s self-perception of subjective need, yielding important results that may contribute to the referral of patients with malocclusion for treatment at public orthodontic health units since it was observed that Orthodontics Protocol used by SUS in Belo Horizonte city does not make it clear which children diagnosed with malocclusion should be referred to the orthodontic specialty. General clinical dentists can interpret the protocol in different ways so that children will not have the same opportunities to be referred to orthodontic treatment. To change the current reality, a standardization of the protocol must take place and this can be done by examining the children for the diagnosis of malocclusion using an orthodontic index combined with the children’s self-perception of subjective need.

One of the advantages of combining children’s self-perception with the clinical assessment made by the health professional lies with the definition of cases that are more likely to have a negative impact on quality of life, given that, according to some studies,^10^ ^,^ ^11^ there are different psychosocial implications for a single type of malocclusion. 

## CONCLUSIONS

» Children diagnosed with extremely severe malocclusion and those with a pronounced maxillary anterior overjet experienced a larger impact on their quality of life.
» Malocclusion, especially in the anterior teeth, can compromise a child’s psychosocial well-being.
» Further studies are needed with children addressing the impact of malocclusion in the mixed dentition.
